# The Changes of Cerebral Morphology Related to Aging in Taiwanese Population

**DOI:** 10.1371/journal.pone.0055241

**Published:** 2013-01-24

**Authors:** Hsiao-Lan Sharon Wang, Rongjun Yu, Yu-Tzu Wu, Wen-Yuan Lee, Ming-Fan Lin, Chia-Yuan Chen, Ein-Yiao Shen

**Affiliations:** 1 Department of Special Education, National Taiwan Normal University, Taipei, Taiwan; 2 School of Psychology and Center for Studies of Psychological Application, South China Normal University, Guangzhou, China; 3 Institute of Public Health, University of Cambridge, Cambridge, United Kingdom; 4 China Medical University Hospital Taipei Branch, Taipei, Taiwan; 5 Graduate Institute of Acupuncture Science, China Medical University, Taichung, Taiwan; University of Jaén, Spain

## Abstract

A cross-sectional study with the 3-dimensional (3D) MRI reconstruction technique was conducted to investigate cerebral complexity changes related to age differences in native Taiwanese population. In our sample of 85 participants aged between 25 and 81, age was associated with gradual ventricular expansion. A nonlinear quadratic relationship between white matter volume and age was found overall in the brain. Widespread age-related reduction in white matter was detected from late adulthood onwards. However, no significant age-related changes in the cortex and whole brain volume were determined throughout adulthood. These findings provided information in describing brain structural complexity, which might in the future serve as an objective diagnostic index or as a predictive parameter for neurological diseases. Our method then may be used for cross-cultural longitudinal studies to evaluate the effect of disease, environment and aging on the brain.

## Introduction

Every living creature is susceptible to aging. According to demographics, in many modern societies, the improvement of human health care has been accompanied with an increase of life expectancy, whereby a great proportion of individuals aged over 60 in developed countries [1]. Similarly, the percentage of native elder people in Taiwan has been increasing. Aging has been frequently reported as a significant correlate of the progressive loss of cognitive function, which may be a concern of gradual decay of physiological and neural mechanisms. Seeking to understand the biology of aging and the many disease processes that hinder cognition among the elderly population, recent imaging studies of brain aging have begun to examine the effect of age to the brain structure, predominantly in Caucasian populations. Nevertheless, a variety of factors that differ across ethnic groups, such as diet environmental factors and genetic makeup, could affect the aging process and brain functions [2], [3].

In the literature, a multitude of studies have documented the influence of age upon various aspects of brain function and structure [4], [5]. As for the age-related change of cerebral structure, the shrinkage of cortical regions, such as frontal lobes and parietal lobes, as well as hippocampus, caudate, and putamen, were consistently found in many articles [6], [7], [8], [9], [10]. But age-related gray matter (GM) reductions are not frequently found in limbic/paralimbic structures [11], [12]. Some subcortical areas of the brain have demonstrated a greater diminished volume along with age [13]. Autopsy studies indicate that brain weight in both men and women declines by at least 10% between the ages of 25 and 75+ years [14], [15]. After the age of 50, volume decreases at the rate of about 2% per decade and the gray/white ratio increased, indicating gray matter volume may be decreasing while white matter volume may be increasing [16]. This age-dependent modification in structure may occur as a result of significant atrophy of cerebral cortex, extensive loss of grey matter, paired with an expansion of ventricular spaces, which might be rendered as an aged brain “smaller” in size. However, whether the “smaller” aged brain is caused by ventricular expansion or grey matter losses or the combination of both factors still need further exploration. Also, previous studies mainly use Caucasian population and little is known whether the same patterns exist in the native Asian population, who constitute the most rapidly aging population grouping in the world. For example, adults over the age of 65 years represented only 4.9% of the Chinese population in 1982 [17], but increased to 6.96% of 1.3 billion in 2000 (National Bureau of Statistics People's Republic of China, 2001).

Moreover, a controversial influence of gender on the age-related trends of neural structural complexity and cortical sulcus was of interest in several studies. For example, Blanton et al. [18] found a gender-related alteration in the left superior frontal and right inferior frontal regions, as estimated by fractal dimension (FD) or complexity of sulcal/gyral convolutions, with complexity significantly increasing in females with age. Also Luders et al. [19] found greater gyrification in women than men in frontal and parietal regions across different ages. However, confronting with this funding, a previous study by Free et al. [20] suggested an equal convolution of cerebral cortex and white matter surface among males and females. It appears inconsistent while some MRI based studies showing a slower decline in the gray matter in women [21], [22], some others could not find significant sex differences in brain aging [23], [24]. It is thus still unclear whether significant aging differences existed between genders or not.

In this study, a cross-sectional design across 56 years (i.e., 25 to 81 years old) was conducted to investigate the age-related cerebral adaptation in 85 healthy native Taiwanese adults. The voxel based analysis by a novel imaging technique (e.g., three dimensional magnetic resonance images; 3D MRI) was employed to capture multiple aspects of aged-brain structural characteristics in relation to gender. This imaging method may provide a more robust detection than the conventional 2D imaging does, regarding the revelation of subtle variations (see below for technical details). We attempt to systematically measure the age-related modification of 3D cerebral skeletons, surfaces, and general structures in normal aging and explore non-linear patterns that age-related structure changes may follow. The issue of potential effects of gender and disease in this particular population was also discussed.

## Materials and Methods

### Participants

Participants were screened and recruited from the database of the China Medical University Hospital, Taichung, Taiwan. Data of eighty-five adults aged between 25 and 80 years (mean age ± standard deviation  = 47.37±12.22 years) were reported here. There were 48 males (47.31±12.32 years) and 37 females (47.45±12.27 years). They were all right-handed and native Taiwanese people with a similar socioeconomic status (middle class). They use Chinese Mandarin as their first official language and speak the native Taiwanese dialect for their daily communication. Their mean education duration was 14.2±2.1 years. The average IQ of our 85 participants assessed by the Chinese version of Wechsler Abbreviated Scale of Intelligence™ (WASI™) was 106.1±8.1. None of them has history of neurological diseases, any substance dependence, or clinically significant head trauma. Written informed consent was obtained from all individual participants, and all of the research procedures and ethical guidelines were followed and approved by the Institutional Review Board (IRB) of China Medical University.

### Collection of 3D MR images

All subjects underwent structural and 3D MRI scan in a single session using a G.E. 1.5 T Excite MRI machine. A thirty-two channel head coil was used as the RF signal receiver. Sponges were used to fix subjects head within the coil to prevent motion artifact. All images were acquired parallel to anterior-commissure-posterior-commissure line with an auto-align technique. The total scan time for each subject was about 10 minutes.

A 3 Dimensional Spoiled Gradient Recalled Echo (3D SPGR) sequence was used to acquire a whole brain high-resolution T1-weighted MR image in a coronal view. The sequence parameters were TR/TE  = 3300 ms/3 ms, image matrix size  = 256×192×512, bandwidth  = 15.63 kHz, spatial resolution  = 1×1 mm^2^, field of view (FOV)  = 220×220 mm^2^, flip angle  = 35°, and slice thickness  = 1.5 mm without gap. The scanning time is about 3 min.

The MRI unit database transmitted the scans to an established workstation for algorithmic 3D image reconstruction. We used the 3D Amira software system (version 3.1.1, Mercury Computer Systems, Inc., USA) for image processing. Its “brush” and “wrapper” tools enabled us to identify the regions of interest and remove the skull component of the brain with an arithmetic module that isolates the cerebrum component.

**Figure 1:**
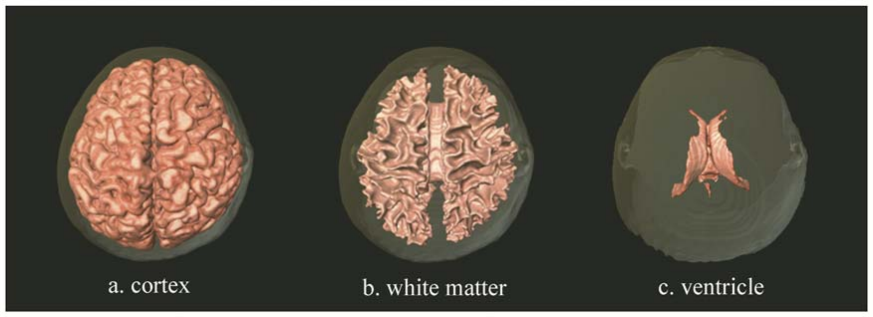
Three-dimensional reconstruction neuroimages of brain at different structures (vertical view).

We limited the gray scale values to 110–155 of 1024 (2^10^) scales in order to approximate the boundary of the grey matter. The software's “threshold” and “edge detection” tools allowed for a precise delineation of the grey matter. Certain areas of less than 50 pixels (area <0.1 cm^2^) were removed to eliminate erroneously identified grey matter. We then repeated the procedure for the white matter with gray scale values in the range of 75–95 of 1024 (2^10^). Subsequently, we computed volumetric measurements of the grey matter with the following formula: Volume of cortex  =  (number of voxels within cortex) × (volume per voxel). This formula can be modified to compute the white matter and whole brain volumes [25], [26], [27]. The MRI scans for 3D image reconstruction of our sample was demonstrated by Figure 1. We then computed brain volume measurements for each participant. The results obtained from the analysis were summarized and reported in the following section.

### Statistical analysis

The first step for data analysis is to screen the dataset, including an examination of variable means and standard deviations, a check for outliers, and review of the distribution of values and errors to ensure that the data were appropriate for the planned statistical analyses. Participants were divided into four age groups (e.g., less than 40, 40–49, 50–59 and more than 60) and the descriptive analysis of their brain volume was shown on Table 1. The distributions of the three measurements of brain volume (cortex, white matter and whole brain) were demonstrated and their overall relationships with age were examined by one-way ANOVA. In the Table 2, the regression modeling was used to investigate the effect of age on brain volume in detail. There are three models included in each measurements of brain volume. All the models are adjusted for sex. The continuous age was included in the Model 1 (linear model) whereas in the Model 2 (non-linear model), the different categorical age group was entered, and likelihood ratio test is used to compare goodness of fit of linear and non-linear models. Finally in the Model 3 (quadratic model), the quadratic age was entered to further explore the non-linear associations between age and several measurements of brain volume. All of the statistical analysis was conducted by Stata 10.1 and SAS 9.1.3.

**Table 1:** Descriptive data of brain morphology.

		Cortex	White matter	Ventricle	Whole brain
	N	Mean (Std.)	Mean (Std.)	Mean (Std.)	Mean (Std.)
Overall	85	662.67 (90.00)	417.99 (72.84)	30.53 (11.38)	1111.19 (125.29)
Age groups					
Age<40	24	682.10 (86.71)	385.85 (55.21)	25.40 (8.31)	1093.36 (128.08)
40< = Age<50	19	649.04 (90.71)	438.82 (73.25)	28.08 (9.42)	1115.94 (132.48)
50< = Age<60	34	655.78 (93.11)	436.71 (75.90)	31.84 (9.32)	1124.34 (117.93)
60< = Age	8	666.07 (91.81)	385.41 (71.22)	46.09 (17.27)	1097.56 (146.9)
p-value (ANOVA)		0.63	**0.015**	**<0.001**	0.81
Sex					
Male	48	705.08 (86.63)	436.02 (69.18)	33.65 (12.55)	1174.75 (105.68)
Female	37	607.66 (59.96)	394.61 (71.67)	26.47 (8.15)	1028.73 (98.35)
p-value (t-test)		**<0.001**	**0.009**	**0.003**	**<0.001**

Note Std.: standard deviation.

**Table 2:** Regression models.

	Cortex	White matter	Ventricle	Whole brain
	Coefficient (95% CI)	Coefficient (95% CI)	Coefficient (95% CI)	Coefficient (95% CI)
**Model 1 (linear model)**				
Age	−0.73 (−2.08, 0.62)	0.67 (−0.57, 1.92)	0.49 (0.33, 0.65)	0.43 (−1.40, 2.26)
Sex (ref.^a^: female)	97.31 (64.18, 130.45)	41.51 (10.95, 72.06)	7.26 (3.31, 11.21)	146.08 (101.23, 190.93)
**Model 2 (non-linear model)**				
<40 (n = 24) (ref.)	–	–	–	–
40∼49 (n = 19)	−18.44 (−65.74, 28.86)	59.69 (18.94, 100.44)	3.86 (−1.84, 9.56)	45.12 (−18.11, 108.34)
50∼59 (n = 34)	−22.76 (−63.61, 18.08)	52.49 (17.31, 87.68)	6.73 (1.80, 11.65)	36.46 (−18.14, 91.05)
≧60 (n = 8)	−3.95 (−66.61, 58.71)	5.11 (−48.87, 59.09)	21.66 (14.11, 29.22)	22.82 (−60.93, 106.58)
Sex (ref.: female)	96.67 (62.93, 130.41)	44.47 (15.40, 73.54)	7.79 (3.72, 11.86)	148.92 (103.82, 194.03)
p-value (LRT ^b^)	0.61	**0.005**	**0.02**	0.43
**Model 3 (quadratic model)**				
Age	0.11 (−7.68, 7.91)	9.85 (2.96, 16.74)	−0.74 (−1.63, 0.14)	9.22 (−1.15, 19.59)
Age^2^	−0.01 (−0.09, 0.07)	−0.09 (−0.16, −0.02)	0.013 (0.004, 0.022)	−0.09 (−0.19, 0.01)
Sex (ref.: female)	97.34 (64.00, 130.68)	41.78 (12.32, 71.24)	7.22 (3.43, 11.02)	146.35 (102.01, 190.68)
p-value (Age^2^)	0.83	**0.01**	**0.005**	0.09

a. ref.: reference group.

b. LRT: likelihood ratio test, for testing the non-linear the association between age and dependent variables.

## Results

Descriptive results of average brain volume were summarized in Table 1. For all of our participants (n = 85), the average volume of cortex was 662.67 cm^3^ (SD  = 90.00 cm^3^), the average volume of white matter was 417.99 cm^3^ (SD  = 72.84 cm^3^), and the average volume of ventricle was 30.53 cm^3^ (SD  = 11.38 cm^3^). Their average whole brain volume was 1111.19 cm^3^ (SD  = 125.29 cm^3^). Planned pairwise comparisons were carried out to investigate the potential impact of age upon the average brain volume. As can be seen, there was a significant group difference in the average volume of white matter (p = .015) and of ventricle (p<.001); however, no significant group differences were found in the average volume of cortex and whole brain. Male subjects demonstrated greater average volumes than the female group in the cortex, white matter, and whole brain.

In the Table 2, we found that three of the four brain volume measurements were not significantly associated with age. Only the ventricle was significantly associated with age and the volume increased 0.49 (95% CI: 0.33, 0.65) per year. The results indicated that there were no substantial linear relationships between age and the measurements of brain volume.

In the Model 2, the significant p-values of likelihood ratio test in volume of white matter and ventricle indicated their non-linear associations with age. People with the average age of 40–49 had significantly larger white matter volume (59.69 cm3; 95% CI: 18.94, 100.44) compared to people with the average age less than 40. People aged 50–59 had significantly larger whiter matter volume (52.49 cm3; 95% CI: 17.31, 87.68) than the baseline group. The white matter volume of elder age group (≧60 years old) was larger (5.11 cm3; 95% CI: −48.87∼ 59.09) than which of the people less than 40, although it did not reach the statistically significant level. People with the average age of 40–49 had slightly larger ventricle volume (3.86 cm3; 95% CI: −1.84, 9.56) compared to people with the average age less than 40. People aged 50–59 had significantly larger ventricle volume (6.73 cm3; 95% CI: 1.80, 11.65) than the baseline group. The ventricle volume of elder age group (≧60 years old) was significantly larger (21.66 cm3; 95% CI: 14.11, 29.22) than the youngest age group. There were no significant associations between age groups, cortex and whole brain volume.

**Figure 2:**
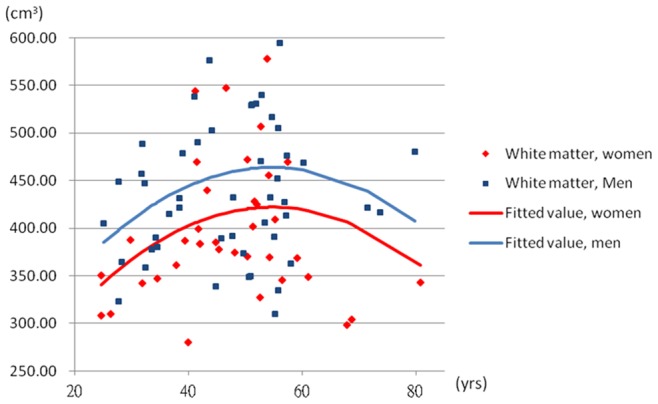
The relationship between age and average white matter volume. The volume of white matter  = −0.09× age^2^+9.85× age+41.76× sex+152.56; male  = 1, female  = 0.

**Figure 3:**
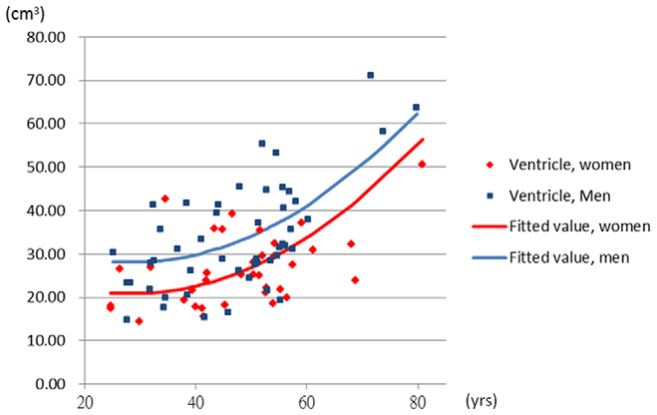
The relationship between age and average ventricle volume. The volume of ventricle  = 0.013× age^2^−0.74× age+7.23× sex+31.4; male  = 1, female  = 0.

**Figure 4:**
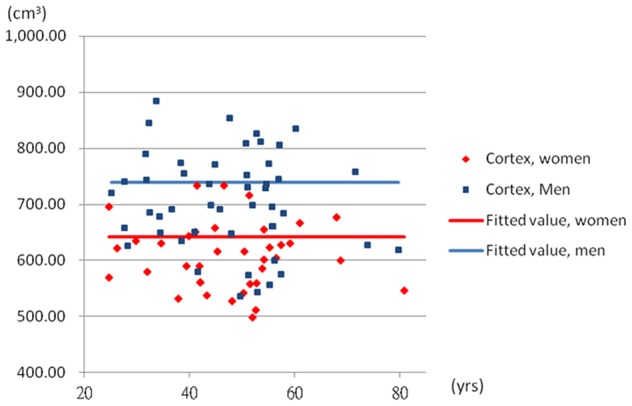
The relationship between age and average cortex volume. The volume of cortex  = 642.37+97.31× sex; male  = 1, female  = 0.

**Figure 5:**
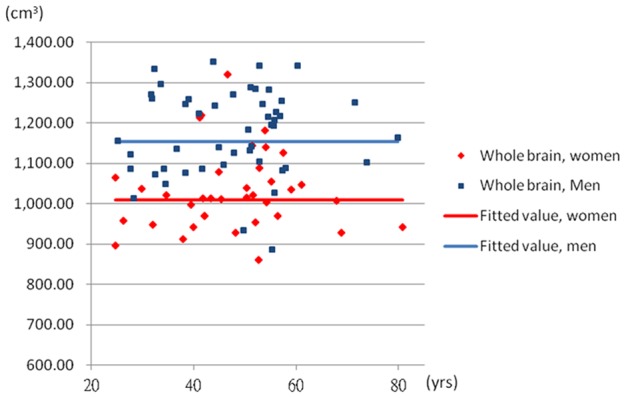
The relationship between age and average whole brain volume. The whole brain volume  = 1008.13+146.08× sex; male  = 1, female  = 0.

To further explore the non-linear associations, the quadratic age term was introduced into the Model 3. The coefficients of age were 9.85 (95% CI: 2.96, 16.74) in the white matter and −0.74 (95% CI: −1.63, 0.14) in the ventricle. The coefficients of quadratic age were −0.09 (95% CI: −0.16, −0.02) in the white matter and 0.013 (95% CI: 0.004, 0.022) in the ventricle when the sex was adjusted. The Figure 2 and 3 demonstrated the quadratic model of age in the white matter and ventricle. The Figure 4 and 5 showed the likely corresponding between age and volume in the cortex and whole brain.

To examine the potential statistic impact of few elders on our results, we also presented the regression models of data excluding the 8 subjects over 60 years old (see Table S1). We found that the effect of quadratic age on the white matter and ventricle volumes was not significant. However, the volume of white matter increased largely in the age group of 40–49 years old and decreased slightly after 50 years old (Figure S1). In the white matter, the non-linear model was still significant, *p* = .03. The quadratic model of age in the white matter approached the significant level, *p* = .07. On the other hand, the association between age and ventricle appeared more likely to be linear instead (Figure S2). To a certain extent, the findings were partly consistent with our prior report of all participants.

Concerning the gender differences, we found that the brain volume of male participants was significantly higher than female in all the models. The modified effect of sex was examined in the regression models but the interaction term did not importantly explain the associations between age and the change of brain volume in Taiwanese population. The pattern of brain volume with age in male and female participant was more likely to be parallel.

## Discussion

Our study was set to analyze 3D MRI data for detection of cerebral structural adaptations related to age in Taiwanese population. The study comprehensively evaluates the volumetric change of general structure, cortical surface, white matter and cerebral ventricle regarding age and gender differences. Our 3D volumetric fractional analysis demonstrated that there was a morphometric difference of white matter and ventricle betweens groups. Elder subjects tended to have a higher volume of ventricle. There was a positive correlation between age and the volume of ventricle, although this relationship may not be linear. This change of the volume of ventricle related to aging might be described by a quadratic function. As for the variability of white matter structure in normal aging, the change of white matter was associated with quatartic age. The sample distribution appears to be the parabola which was opened downward. The volume of white matter enlarged gradually with age around 50 years old and started to decline with the increase of age afterward. Subjects at the middle age (e.g., 50–59 years old) tended to have a higher volume of white matter. No significant age-related volume differences were found in the cortex and general structure (whole brain). With respect to the potential gender effect, our results showed that men had higher volumes than women for overall measures of brain volume (e.g., cortex, white matter, ventricle and whole brain). The aging pattern of cerebral white matter and ventricle volumes was similar across men and women, suggesting little effect of gender upon the age-related change of cerebral morphology.

The present data were consistent with much recent neuroimaging research in Caucasian population which has brought reports regarding white matter changes in elder people, suggesting a process of dendrite and dendritic spine loss and shrinkage of axons and myelin [28], [29]. For instance, by a DTI study, the relationship between total white matter volume and age shows a nonlinear quadratic interaction, which is consistent with our 3D MRI results [30]. Interestingly, we found that the increasing volume of white matter found in the early adulthood may coherent with the progress of myelinization of neural structure beyond adolescence in Taiwanese population too On the other hand, the degeneration of WM we observed from the fifth decade was consistent with several VBM-based studies previously [21], [31]. At a microscopic level, axonal and myelin thinning may decrease conduction velocity, which reduces the information processing efficiency and increases the reaction time. As a result, across nations, peoples and cultures, aging may influence rate and amount of motor activity similarly, as well as the proficiency of some cognitive function, such as memory retrieval [32], [33]. Another structural feature significantly affected by the aging process is the ventricle. The age-related modification in the volume of ventricle may imply that the previously reported age increases in CSF are associated with this expansion. As the ventricular dilatation and brain atrophy has been found related to some neurological and psychiatric disorders (e.g. Alzheimer's disease and depression), future studies may design a more sensitive measure of the time course of white matter and ventricular change for clinical inspection.

In contrast, the changes of cortical and whole brain volume appear less evident across groups, suggesting a slight association between gray matter volume and age. The findings were unexpected because previous research indicated substantial age-related volume reduction in gray matter and whole brain due to the genuine decreases in neurons and synapses [34], [35]. This does not mean, however, that the grey matter was affected with no consequence to age, but perhaps that such consequences were manifested by signal transmission changes within matter rather than shrinkage. Also the native, cultural or environmental effect upon this inconsistent finding may be worth of exploration in the coming research. In addition, future study may include some complementary analyses (e.g. fractional anisotropy) of advanced MRI techniques to better understand the minute alteration related to age in the brain.

Some limitations in our study are worth mentioning. First, the sample size in this study is relatively small, especially for subjects of age more than 60, which may result in Type 2 error and. A scarce representation of individuals after the sixth decade may limit the generalization of our conclusions to older population (age >60). More work will, however, be required to investigate those relationships using a large number of subjects over 60 years of age. Second, although the general IQ (i.e. WASI™) was measured in the current study, it is still unclear how the age related changes of cerebral morphology are correlated with specific cognitive declines, such as cognitive control, working memory, and reasoning. For future studies, it would be interesting to examine the cerebral morphology related to specific cognitive functions as measured by classic paradigms including stoop task, go-nogo task, working memory task and so on. Third, the cross-sectional design employed in the present study has an inherent limitation. In cross-sectional design, age effect is inferred from measurements made from subjects of different ages, which may bias results due to potential cohort differences. This is especially important considering the dramatic political and economic changes happened in Taiwan. However, longitudinal designs, where each subject serves as his or her own control, are also more difficult to conduct and may suffer from other issues. Finally, it is worth noting that our sample is the native Taiwanese people. Future studies may directly compare age based cerebral morphology changes across samples (e.g. Caucasian population, Taiwanese population, and other Asian population) to explore culture influences on aging.

In conclusion, we measured the cerebral structures to characterize the spatial and temporal pattern of age-related changes that occur in cortex and white matter, specifically in the native Taiwanese people. Our group results reported the significant nonlinear (quadratic) relationships between white matter volume and age. The volume increase of ventricular space was associated with age as well. However, no significant cortical change was found related to age. Our 3D MRI study benefited from some imaging advances in vivo with morphometric analyses compared to previous dissection studies investigating the ageing brain, although there are some limitations. For example, we applied a cross-sectional design and the study recruited different cohort of participants in different age groups. The condition may diminish the power and sensitivity to detect the cerebral changes across the age range in normal subjects. Also, the age clustering in this study was arbitrary, although the grouping selection was broadly in line with prior research. Thus a longitudinal study to address these questions with neuropsychological evaluations is currently underway. Participants' physical and psychological measures are documented year by year. Cultural and native factors of individuals are carefully traced. Nevertheless, the observed aging patterns and dynamics in the neural mechanism we reported in this study may provide implications for prospective studies on some particular neurodegenrative diseases in this native, such as Huntington's Disease. The association between age and cerebral morphology might be also explained by some common diseases they have, such as alcohol addition, diabetes and hypertension. These factors might cause bidirectional modified effect on the relationship between ageing and cerebral morphology. The application of our 3D MRI technique may be helpful to improve our understanding of neural substrates underlying the typical and untypical aging progress across nations.

## Supporting Information

Figure S1 **The relationship between age and average white matter volume (n = 77).** The volume of white matter  = 0.17× age^2^+15.98× age+32.96× male+34.08.
(TIF)

Figure S2 **The relationship between age and average ventricle volume (n = 77).** The volume of ventricle  = 0.32× age+5.85× male+11.30.
(TIF)

Table S1 **Regression models (n = 77).**
(DOCX)
