# Supplementary figures and images for: The Changes of Cerebral Morphology Related to Aging in Taiwanese Population
Source: PLoS One. 2013 Jan 24;8(1):e55241. doi: 10.1371/journal.pone.0055241 (PMC3554665; doi:10.1371/journal.pone.0055241)

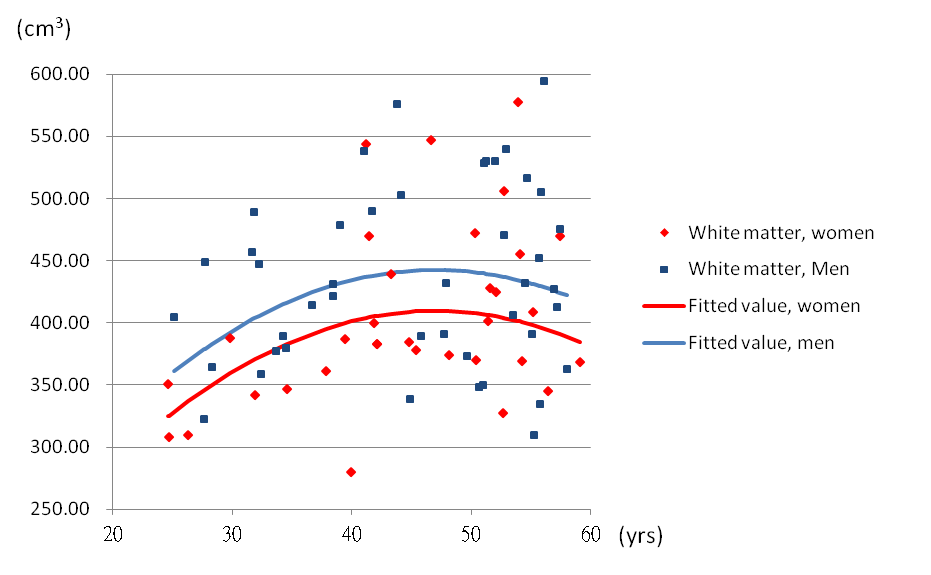

Supplement: Figure S1 — The relationship between age and average white matter volume (n = 77). The volume of white matter = 0.17× age2+15.98× age+32.96× male+34.08. (TIF) [file pone.0055241.s001.tif]

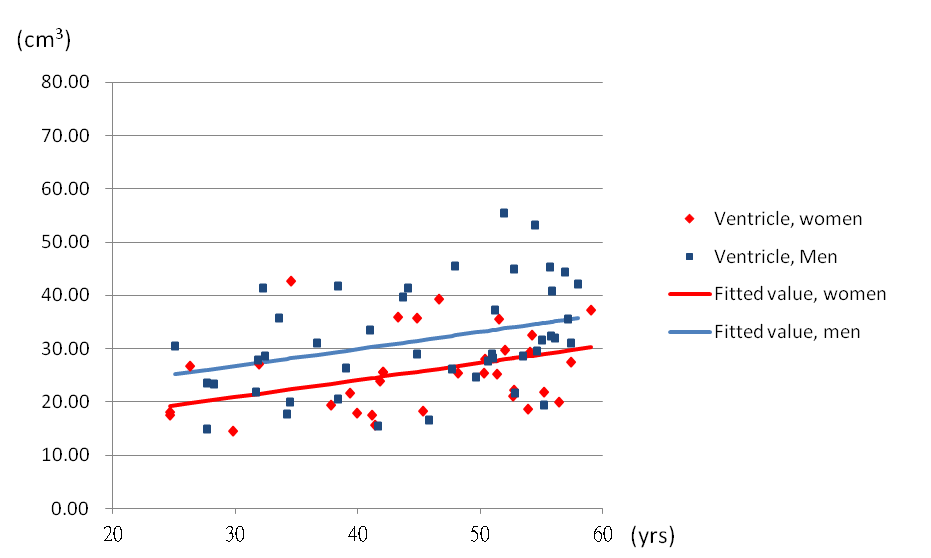

Supplement: Figure S2 — The relationship between age and average ventricle volume (n = 77). The volume of ventricle = 0.32× age+5.85× male+11.30. (TIF) [file pone.0055241.s002.tif]
