# Supplementary material for: The Changes of Cerebral Morphology Related to Aging in Taiwanese Population
Source: PLoS One. 2013 Jan 24;8(1):e55241. doi: 10.1371/journal.pone.0055241 (PMC3554665; doi:10.1371/journal.pone.0055241)
Supplement: Table S1 — Regression models (n = 77). (DOCX) [file pone.0055241.s003.docx]

Table 1S Regression models (n = 77)

|  | White matter | Ventricle |
| --- | --- | --- |
|  | Coefficient (95% CI) | Coefficient (95% CI) |
| **Model 1: linear** |  |  |
| Age | 1.93 (0.33, 3.53) | 0.32 (0.12, 0.51) |
| Sex (ref.^a^: female) | 31.15 (-0.59, 62.90) | 5.85 (2.00, 9.71) |
| **Model 2: non-linear** |  |  |
| <40 (n=24) (ref.) | -- | -- |
| 40~49 (n=19) | 58.41 (16.83, 99.99) | 3.61 (-1.66, 8.88) |
| 50~59 (n=34) | 52.18 (16.29, 88.07) | 6.67 (2.11, 11.22) |
| Sex (ref.: female) | 36.01 (4.81, 67.21) | 6.12 (2.16, 10.08) |
| p-value (LRT ^b.^) | **0.03** | 1.00 |
| **Model 3: quadratic** |  |  |
| Age | 15.98 (0.71, 31.25) | 0.16 (-1.74, 2.06) |
| Age^2^ | -0.17 (-0.34, 0.01) | 0.002 (-0.02, 0.02) |
| Sex (ref.: female) | 32.96 (1.65, 64.27) | 5.83 (1.94, 9.73) |
| p-value (Age^2^) | 0.07 | 0.87 |

^a.^ ref.: reference group

^b.^ LRT: likelihood ratio test, for testing the non-linear the association between age and dependent variables.
